# Technological and Modelling Progress in Green Engineering and Sustainable Development: Advancements in Energy and Materials Engineering

**DOI:** 10.3390/ma16227238

**Published:** 2023-11-20

**Authors:** Jaroslaw Krzywanski, Agnieszka Kijo-Kleczkowska, Wojciech Nowak, Marcio L. De Souza-Santos

**Affiliations:** 1 Department of Advanced Computational Methods, Faculty of Science and Technology, Jan Dlugosz University, 13/15 Armii Krajowej, 42-200 Czestochowa, Poland; 2 Faculty of Mechanical Engineering and Computer Science, Czestochowa University of Technology, Dabrowskiego 69, 42-201 Czestochowa, Poland; a.kijo-kleczkowska@pcz.pl; 3 Faculty of Energy and Fuels, AGH University of Science and Technology, Mickiewicza 30, 30-059 Krakow, Poland; wnowak@agh.edu.pl; 4 Department of Energy, School of Mechanical Engineering, UNICAMP—University of Campinas, Campinas 13083-970, SP, Brazil; dss@csfmb.com

**Keywords:** sustainability, net-zero emissions, energy efficiency, waste-to-energy, fuels, modelling, optimization, artificial intelligence, bio-inspired methods, simulation, complex systems

## Abstract

Due to a growing number of environmental issues, including global warming, water scarcity, and fossil fuel depletion, the topic of modern materials in energy is becoming crucial for our civilization. The technological advancements that have been observed bring many innovations that significantly impact how energy can be generated, stored, and distributed. Moreover, new opportunities have emerged in energy and materials engineering due to the increasing computational capability of current data processing systems. Methods that are highly demanding, time-consuming, and difficult to apply may now be considered when developing complete and sophisticated models in many areas of science and technology. Combining computational methods and AI algorithms allows for multi-threaded analyses solving advanced and interdisciplinary problems. Therefore, knowledge and experience in this subject, as well as the investigation of new, more efficient, and environmentally friendly solutions, currently represent one of the main directions of scientific research. The Special Issue “Advances in Materials: Modelling Challenges and Technological Progress for Green Engineering and Sustainable Development” aims to bring together research on material advances, focusing on modelling challenges and technological progress (mainly for green engineering and sustainable development). Original research studies, review articles, and short communications are welcome, especially those focusing on (but not limited to) artificial intelligence, other computational methods, and state-of-the-art technological concepts related to the listed keywords within energy and materials engineering.

## 1. Introduction

Observed climate changes, environmental pollution, the shortage of drinking water, growing demands for energy, and the simultaneous depletion of natural resources force the search for new technologies, including modern materials, which are key to sustainable development. The goal is sustainable development, striving for zero-emission technologies and increasing the energy efficiency of devices. These efforts are accompanied by requirements for selecting and testing new materials.

This work aims to encourage the scientific community to submit work aimed at meeting the above challenges, considering modelling issues and practical technological aspects.

The exemplary areas and topics for possible contributions are listed in the next section.

## 2. Advancements in Energy and Materials Engineering

In the era of the observed climate change, such clan coal technologies, waste heat application methods, and effective energy conversion systems play a crucial role. However, clean coal technologies and carbon capture and storage pose challenges centered around the efficiency and quality of the materials used [1,2].

Innovations in carbon capture technologies (CCTs), including materials used for CO_2_ capture and utilization (CCU) [3,4], artificial-intelligence-driven smart materials [5,6], oxygen carriers [7,8,9,10], sorbents in adsorption cooling and desalination systems [8,9], as well as construction materials of energy devices [11,12,13], play a key role in implementing these technologies on a larger scale and are crucial for achieving the United Nations’ Sustainable Development Goals (SDGs) [14].

Thermal fuel conversion, including clean coal fluidized bed technologies, allows various fuels and wastes, including biomass and alternative fuels, to be used [15]. It is impossible not to mention new solutions in the area of energy storage [16,17,18] and thermomechanical properties, including phase change materials (PCMs) used for thermal energy storage and the modelling of novel composite materials formed by combining PCMs with other materials [19]. In this area, we can also point to innovative approaches using solar energy and heat pumps [20,21,22], ocean [23,24], and wind [25,26].

In pursuit of the goal of net-zero emission [27,28], waste-to-energy (WtE) technology is becoming more and more important, allowing requirements centered around a circular economy, greenhouse gas reduction [29], and sustainable waste management systems [30,31] to be met.

Recent advances in materials science also include new materials used in refrigeration, including emerging materials and rational strategies in passive daytime radiative cooling (PDRC) [32], thermoelectric devices [33], and adsorption chillers [34]. For example, novel adsorbents and their modifications must perform better in adsorption cooling and desalination systems, including when being blended with nanomaterials.

Progress in materials science and engineering would not be possible without advances in modelling. These advances include, among other things, combustion and co-combustion processes in power boilers, including the use of artificial intelligence methods [35,36,37,38]. The issues concern materials, but also the operating conditions and operating strategies of energy devices and systems [39,40,41].

## 3. Conclusions

Technological advancements in materials, especially in the energy sector, bring enormous challenges. They also provide an opportunity to create a more sustainable future. Significant progress can be made and contribute to developing solutions in order to achieve net-zero emissions goals through cooperation and by sharing experience and knowledge.

## Data Availability

Data sharing not applicable.
